# Synthesis and Characterization of Stimuli-Responsive Poly(2-dimethylamino-ethylmethacrylate)-Grafted Chitosan Microcapsule for Controlled Pyraclostrobin Release

**DOI:** 10.3390/ijms19030854

**Published:** 2018-03-14

**Authors:** Chunli Xu, Lidong Cao, Pengyue Zhao, Zhaolu Zhou, Chong Cao, Feng Zhu, Fengmin Li, Qiliang Huang

**Affiliations:** Institute of Plant Protection, Chinese Academy of Agricultural Sciences, No. 2 Yuanmingyuan West Road, Beijing 100193, China; springxcl2013@126.com (C.X.); pengyue_8825@163.com (P.Z.); hbndzzl91@163.com (Z.Z.); ccao@ippcaas.cn (C.C.); gzzbszf@163.com (F.Z.); fmli@ippcaas.cn (F.L.)

**Keywords:** grafted chitosan, pyraclostrobin, microcapsulation, controlled release, photostablity, acute toxicity

## Abstract

Controllable pesticide release in response to environmental stimuli is highly desirable for better efficacy and fewer adverse effects. Combining the merits of natural and synthetic polymers, pH and temperature dual-responsive chitosan copolymer (CS-*g*-PDMAEMA) was facilely prepared through free radical graft copolymerization with 2-(dimethylamino) ethyl 2-methacrylate (DMAEMA) as the vinyl monomer. An emulsion chemical cross-linking method was used to expediently fabricate pyraclostrobin microcapsules in situ entrapping the pesticide. The loading content and encapsulation efficiency were 18.79% and 64.51%, respectively. The pyraclostrobin-loaded microcapsules showed pH-and thermo responsive release. Microcapsulation can address the inherent limitation of pyraclostrobin that is photo unstable and highly toxic on aquatic organisms. Compared to free pyraclostrobin, microcapsulation could dramatically improve its photostability under ultraviolet light irradiation. Lower acute toxicity against zebra fish on the first day and gradually similar toxicity over time with that of pyraclostrobin technical concentrate were in accordance with the release profiles of pyraclostrobin microcapsules. This stimuli-responsive pesticide delivery system may find promising application potential in sustainable plant protection.

## 1. Introduction

Pesticides are widely used to improve the production of major crops to meet the global food demand of the escalating population and ensure sustainable development of agriculture. Due to environmental conditions and mode of application, conventional pesticide formulations created a series of serious human health problems and global environmental contamination [1]. Controlled-release formulation (CRF) has been a topical subject of research in recent years, which has been widely applied in pesticides worldwide [2]. The technology of CRF of pesticide provides a better solution for the current challenges that modern pesticides face, for it not only could deliver active ingredients slowly and continuously for longer durations to target, but also enhance the efficiency of pesticide utilization and alleviate the adverse effect on the environment [3,4,5,6].

The performance of CRF on controlling the release profile of pesticide is closely related to the carrier materials [7,8]. To this point, the selection of materials used as carrier agents in pesticide formulations is crucial. Due to the excellent biocompatibility, versatile biological activity, and complete biodegradability in combination with low toxicity, chitosan (CS), the second most abundant polysaccharide, is currently being explored intensively for drug targeting, delivery, and release [9,10,11,12]. In agriculture, especially for pesticides, CS also shows promising application potential in delivery systems [13,14,15,16,17,18,19,20,21,22]. However, CS is only soluble in dilute acid solutions, which limits its applications. In addition, it is highly hydrophilic due to abundant hydroxyl and amine groups, which bind strongly with hydrophilic guest molecules through hydrogen bonding and dipolar interactions [23]. This impedes the release of encapsulated cargo molecules. Recently, there has been a growing interest in the chemical modifications of CS in order to improve its solubility as well as the hydrophobicity, which would endow CS with special properties and enlarge its fields of potential applications. As a consequence, the release profiles of hydrophilic cargo molecules can be finely modulated, and the amphiphilic CS carriers could be versatilely fabricated, which can efficaciously encapsulate hydrophobic drugs through self-assembly and sustainably release them. Not long before, we prepared amphiphilic CS-co-poly(lactide) copolymer nanoparticles for encapsulating and releasing hydrophobic insecticide imidacloprid [24]. 

Among various chemical modifications of CS, graft copolymerization of vinyl monomers onto CS has been shown to be a promising method for chemical and mechanical modification to generate novel properties and functions [25]. Free radical polymerization is mostly used as a “grafting from” method to perform CS modification [26,27]. Among these vinyl monomers, poly(2-dimethylaminoethylmethacrylate) (PDMAEMA) copolymers exhibit excellent biodegradability, biocompatibility, pH- and thermo sensitivities, which have attracted great attention currently in controlled or sustained delivery systems [28]. Grafting of PDMAEMA onto CS was demonstrated to have pH-and temperature-responsive characters [27,29,30]. Combining the merits of natural and synthetic polymers, CS-*g*-PDMAEMA block copolymers have promising application potential in the agricultural field, especially for controlled pesticide release to address the current challenges that modern pesticides are facing.

**Figure 1:**
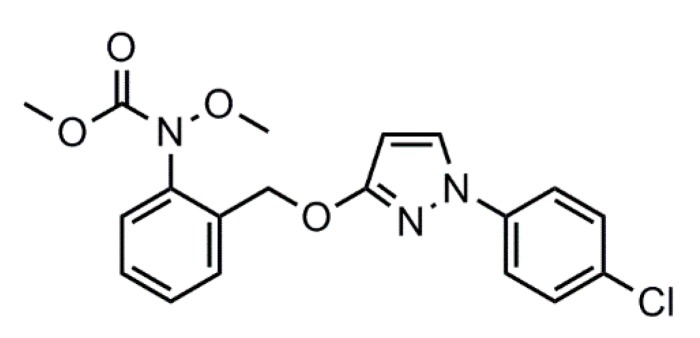
Chemical structure of pyraclostrobin.

Pyraclostrobin, methyl *N*-(2-(1-(4-chlorophenyl)pyrazol-3-yloxymethyl)phenyl) (*N*-methoxy)carbamate (Figure 1) is the second generation of strobilurins developed by Badische Anilin-und-Soda-Fabrik (BASF) in 2000. With broad antifungal activity spectrum and high efficiency, pyraclostrobin had been approved in more than 50 countries for over 100 crops in over 100 indications by the end of 2005 [31]. However, pyraclostrobin has proved to be poisonous to aquatic organisms [32,33], leading to long-term adverse effects on aquatic environments. Furthermore, photolysis and hydrolysis of pyraclostrobin occur easily in water solution. Therefore, it is imperative to develop a method to overcome such drawbacks. Microencapsulation of pyraclostrobin with biodegradable materials would provide an alternative way to break these limitations. 

In the present study, pH- and temperature-responsive CS-*g*-PDMAEMA copolymers were synthesized through free radical polymerization initiated by potassium persulphate (KPS). CS-*g*-PDMAEMA microcapsule loaded with pyraclostrobin was prepared via an emulsion chemical cross-linking method with glutaraldehyde as a cross-linking agent. The parameters influencing the loading content (LC) and encapsulation efficiency (EE) were studied. The effects of pH and temperature on the release profiles of pyraclostrobin were also studied. Moreover, the photostability improvement of pyraclostrobin in microcapsule as well as the acute toxicity against zebra fish was also demonstrated. 

## 2. Results and Discussion

### 2.1. Preparation and Characterization of Pyraclostrobin Encapsulated Microcapsules

**Figure 2:**
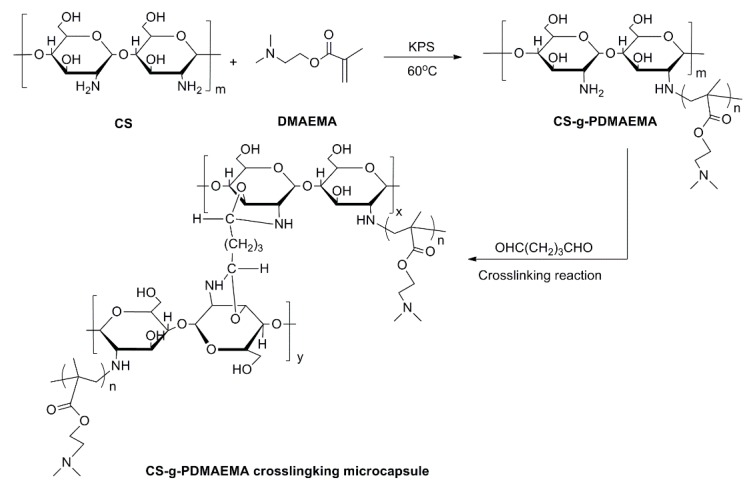
Proposed reaction process for preparation of CS-*g*-PDMAEMA copolymer and microcapsule.

In the present study, free radical polymerization was adopted for modification to endow CS with versatile properties. Due to the excellent biodegradability, biocompatibility, pH- and thermo sensitivities of PDMAEMA copolymers, DMAEMA monomer was used for graft copolymerization of CS initiated by KPS. A CS-*g*-PDMAEMA copolymer could be facilely prepared as described in Figure 2. The positively charged CS-*g*-PDMAEMA has been used as negatively charged plasmid DNA vectors through formation of polyplexes via electrostatic interaction [26]. However, it is not easy to encapsulate hydrophobic pesticide pyraclostrobin by CS-*g*-PDMAEMA copolymer itself through the formation of polymeric micelles via self-assembly, possibly due to the low hydrophobility of PDMAEMA. Taking full advantage of the residual –NH_2_ group in the skeleton of CS, the emulsion chemical cross-linking method with glutaraldehyde as a cross-linking agent was used to expediently fabricate microcapsule in situ entrapping the pesticide, as indicated by Figure 2. 

**Figure 3:**
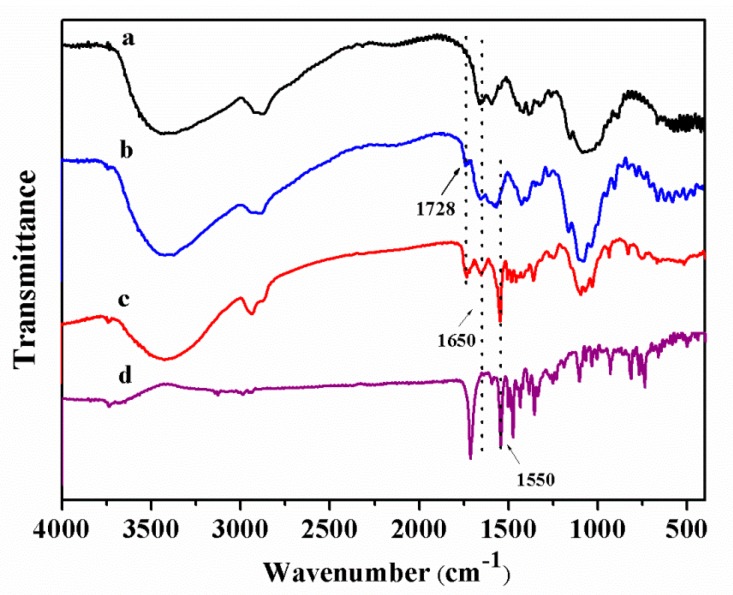
FT-IR spectra of CS (**a**); CS-*g*-PDMAEMA (**b**); pyraclostrobin microcapsule (**c**); and pyraclostrobin (**d**).

**Figure 4:**
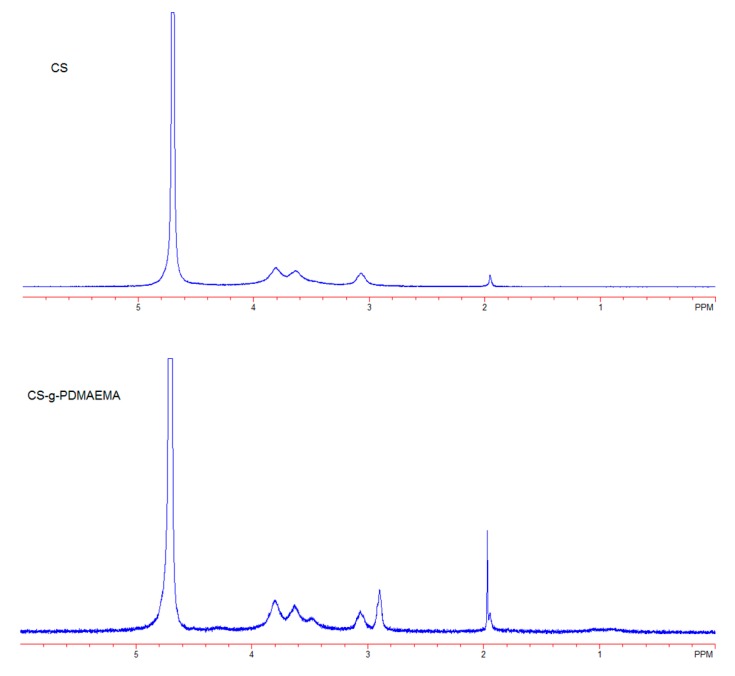
^1^H-NMR spectra of CS and CS-*g*-PDMAEMA.

CS-*g*-PDMAEMA copolymer and pyraclostrobin microcapsules were further characterized. Fourier transform infrared (FT-IR) spectra of CS, CS-*g*-PDMAEMA, pyraclostrobin, and Pyr@CS-*g*-PDMAEMA are shown in Figure 3. The obvious difference between original CS (Figure 3a) and CS-*g*-PDMAEMA (Figure 3b) is the appearance of the absorption band at 1728 cm^−1^ ascribed to C=O stretching, indicating that the copolymerization of CS and DMAEMA occurred [34]. The absorbance peak appearing at 1650 cm^−1^ in Pyr@CS-*g*-PDMAEMA (Figure 3c) can be assigned to imine bonds (–C=N) stretching vibrations, confirming the succsssful cross-linking reaction between amino groups of CS and aldehyde group of glutaraldehyde [35]. Compared to Figure 3d, the characteristic peak of pyraclostrobin appearing at 1550 cm^−1^ in Figure 3c indicated the successful incorporation of pyraclostrobin into microcapsules. The ^1^H-NMR spectra of CS and CS-*g*-PDMAEMA are shown in Figure 4. By comparing the integrated areas of peaks at 3.5–3.9 ppm (m, assigned to glucosamine unit, H-3, H-4, H-5, H-6, H-6′) with the methyl peak at 2.9 ppm adjacent to the tertiary amine in the PDMAEMA side chain, the percentage of DMAEMA in the CS backbone was estimated to be 25%. 

**Figure 5:**
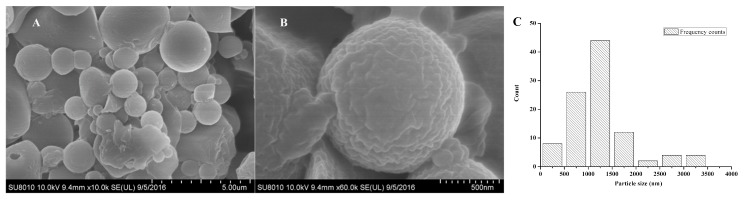
SEM images of pyraclostrobin microcapsules ((**A**) scale bar of 5.0 μm; (**B**) scale bar of 0.5 μm) and the histogram of particle size distribution (**C**).

The morphology and particle size of Pyr@CS-*g*-PDMAEMA were observed by scanning electron microscope (SEM) as shown in Figure 5A,B. The average diameter was 1.2 μm determined by statistical analysis of more than 100 particles. The histogram of particle size distribution of pyraclostrobin microcapsules is indicated in Figure 5C. In addition to the spherical microcapsules with the average diameter of 1.2 μm, some irregular small particles that possibly resulted from the mechanical stress during the stirring process, or the movement of the moisture during the drying period, were also observed [36]. 

**Figure 6:**
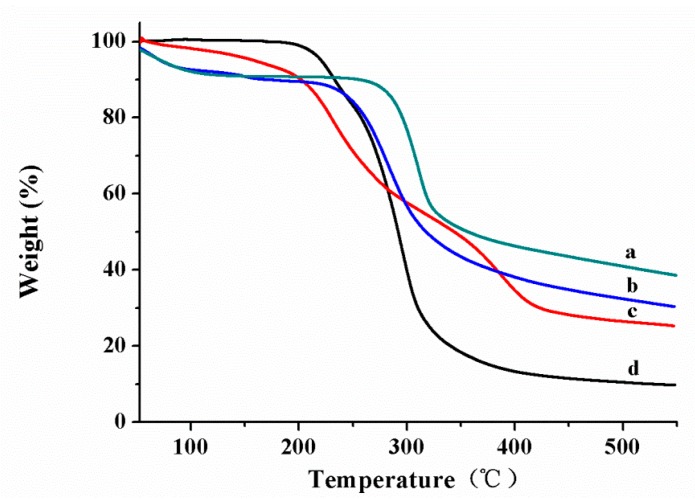
TGA of CS (**a**); CS-*g*-PDMAEMA (**b**); Pyr@CS-*g*-PDMAEMA (**c**); and pyraclostrobin (**d**).

Figure 6 displays the thermogravimetric analysis (TGA) curves of CS (curve 6a), CS-*g*-PDMAEMA (curve 6b), Pyr@CS-*g*-PDMAEMA (curve 6c) and pyraclostrobin (curve 6d). CS-*g*-PDMAEMA has two distinct weight loss stages: the first weight loss at about 50 °C is ascribed to the vaporization of bound water; and the second one started at about 250 °C is due to the thermal decomposition of sample. CS-*g*-PDMAEMA shows increased weight loss than virgin CS, which is attributed to the fact that the PDMAEMA side chain decomposes to a higher degree than the CS backbone under the same conditions. Pyraclostrobin (curve 6c) shows a sharp weight loss that started at about 200 °C due to its decomposition. The three–stage weight loss in Pyr@CS-*g*-PDMAEMA is particularly noteworthy. The first stage loss at about 50 °C is ascribed to the vaporization of bound water; the second weight loss at around 200 °C is decomposition of pyraclostrobin and the third weight loss at about 250 °C is primarily attributed to decomposition of CS-*g*-PDMAEMA, which further proved that CS-*g*-PDMAEMA were loaded with pyraclostrobin. This weight loss trend was similar to those for *N*-carboxyethylchitosan-based poly(*N*,*N*-dimethylamino)ethyl methacrylate graft copolymer. The weight loss increased with the increasing grafting percentages [37]. Moreover, the grafting percentage of CS-*g*-PDMAEMA estimated by TGA data was about 24%. 

### 2.2. Optimization of Loading Content and Encapsulation Efficiency of Pyraclostrobin

With the CS-*g*-PDMAEMA carrier in hand, the LC and EE of pyraclostrobin were next optimized including the ratio of carrier-to-pesticide and the amount of cross-linking agent. As shown in Table 1, the amount of cross-linking agent glutaraldehyde has an important effect on the EE when the ratio of carrier-to-pesticide was kept constant. The EE increased with increasing amount of glutaraldehyde (entries 1–3, Table 1). This is possibly due to the fact that the high mass concentration of cross-linking agent generates a denser macromolecular network in the microcapsule, which can prevent the outward diffusion of the encapsulated pesticide. The carrier-to-pesticide ratio dramatically influenced the LC that was increased with the decreasing ratio (entries 3–5, Table 1). This is possibly ascribed to the higher concentration of pesticide that generated a strong gradient to facilitate the diffusion of cargo molecules into the microcapsules. When the LC and EE were considered together, the carrier-to-pesticide ratio of 1:1 and glutaraldehyde of 1.0 mL were chosen as the optimized conditions. 

### 2.3. Stimuli-Responsive Release of Pyraclostrobin 

**Figure 7:**
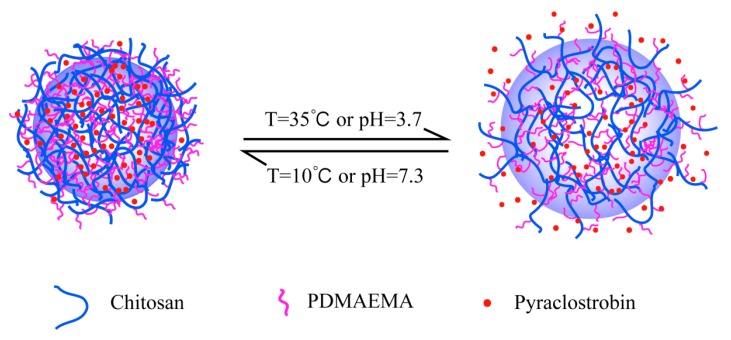
Schematic illustration of responsive release of pyraclostrobin from Pyr@CS-*g*-PDMAEMA microcapsule under different thermo and pH conditions.

Controllable pesticide releases in response to environmental stimuli are highly desirable for better efficacy and fewer adverse effects. According to the dual-response of the carrier chitosan copolymer, the effects of different pH and temperature on the pyraclostrobin microcapsules release were also studied. Figure 7 shows the schematic illustration of the responsive release process of pyraclostrobin from Pyr@CS-*g*-PDMAEMA microcapsule under different thermo and pH conditions.

**Figure 8:**
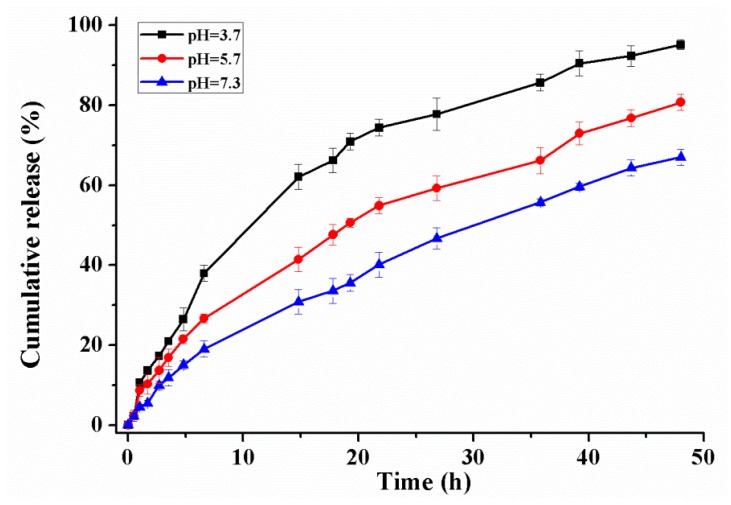
Pyraclostrobin released at different pH value. Error bars correspond to standard errors of triplicate measurements.

The release profile of pyraclostrobin under three different pH values of 3.7, 5.7, and 7.3 at 30 ± 0.5 °C is shown in Figure 8. At pH 3.7, the cumulative release reached up to 40% after the first 7 h, and continuously increased to about 95% after 48 h; at pH 5.7, the cumulative release attained 30% and 80% after 7 and 48 h respectively; at pH 7.3, the cumulative release attained 67% after 48 h. These results clearly demonstrated that the cumulative release of pyraclostrobin was higher in acidic medium in comparison with that in the basic and neutral solution, which is directly proportional to the swelling ratio of the copolymers [38]. As the microcapsules’ exposures to release media, the entrapped pesticide passes into the external receiving medium by crossing the polymeric matrix. The increased acidity of the release medium enhances the protonation of –N(CH_3_)_2_ groups of the grafted polymer, and consequently the intermolecular and intramolecular electrostatic repulsion interactions increase, which facilitates the release of cargo molecules. The similar initial release profiles under different pH values were possibly attributed to the pyraclostrobin near the surfaces of the microcapsules. As time went by, the release rate changed significantly at different pH. The pH-responsive properties of CS-*g*-PDMAEMA were also verified by CO_2_-switchable lower critical solution temperatures and turbidity. These effects are associated with the protonation of the tertiary amine group in PDMAEMA by the H_2_CO_3_ formed in aqueous solution during CO_2_ bubbling [30]. 

**Figure 9:**
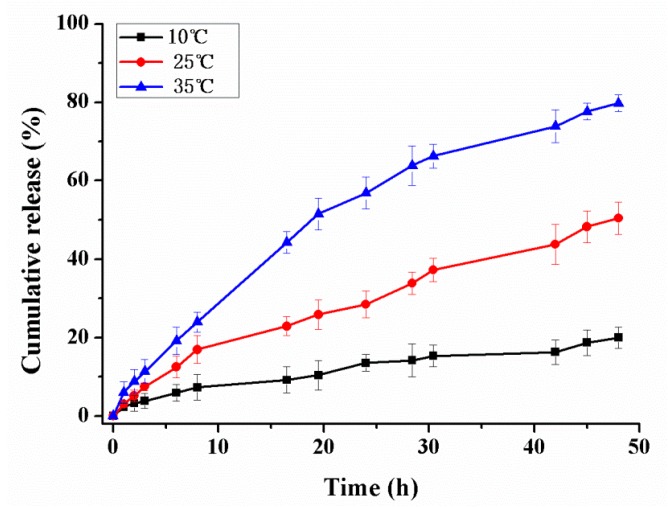
Pyraclostrobin released at different temperature at pH 6.8. Error bars correspond to standard errors of triplicate measurements.

The effect of temperature on the release profiles of pyraclostrobin from microcapsules was also studied at pH 6.8. A tendency of an increased pesticide release with increasing temperature was observed in Figure 9. The cumulative release obtained at 35 °C is higher compared with that at 25 °C and 10 °C. The cumulative release of pyraclostrobin was about 79% at 35 °C, 50% at 25 °C, and 20% at 10 °C after 48 h, respectively. Non-covalent interaction between cargo molecules and carrier material mainly affect the release profile. The major non-covalent interaction between pyraclostrobin and its carrier are Van der Wals and hydrogen bonding, which decrease with the increasing temperature, facilitating the release of pesticide entrapped. On the other hand, the microcapsules are well absorbency swelling with their polymer networks extended at appropriate temperatures. The polymer networks will absorb water molecules until reaching a balance between chemical potential inside and outside the structure. As the temperature decreases, the microcapsules collapse and adopt a compact structure like a hard particle, which finally restricts the pesticide release, as shown in Figure 7. 

### 2.4. Photolysis Kinetics

**Figure 10:**
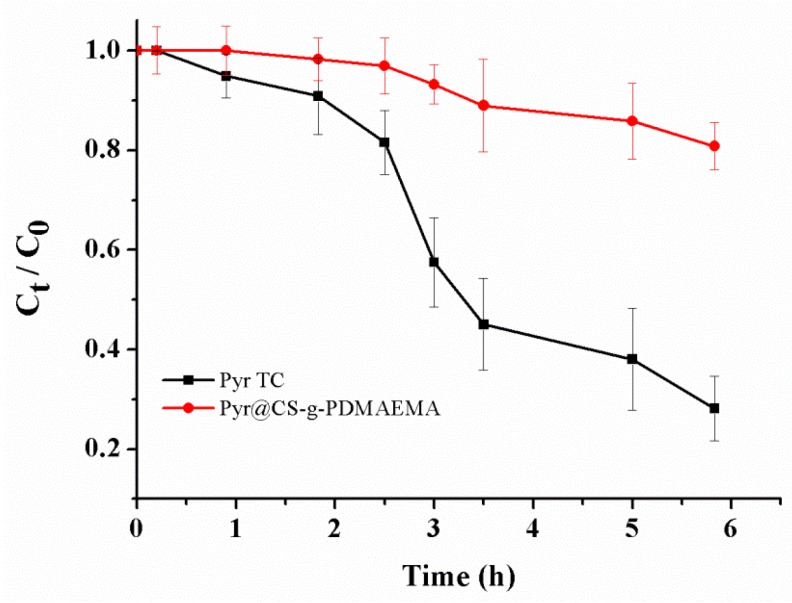
Photodegradation of pyraclostrobin TC and microcapsules under high-pressure mercury lamp.

Pyraclostrobin is unstable under photo irradiation. In order to embody the improved photostability through microcapsulation, the photodegradation of pyraclostrobin microcapsules and 95% pyraclostrobin technical concentrate (TC) as control were investigated under ultraviolet light. The degradation profiles are shown in Figure 10. Photolysis of pyraclostrobin could be characterized by a first-order kinetic equation. As seen in Table 2, in the presence of a high-pressure mercury lamp, the photolytic rate constant (*k*) for pyraclostrobin standard is 0.22 h^−1^, while *k* is 0.03 h^−1^ for microcapsule. The half-life times (*t*_1/2_) of photodegradation for pyraclostrobin TC and microcapsule is 3.2 and 23.1 h, respectively, which clearly indicated the increased photostablity by microcapsulation. Compared to free pyraclostrobin, the improved photostability through microcapsulation is possibly due to the fact that microcapsules reduce the probability of the pesticide in direct contact with the incident light. The carrier material not only blocks out the light, but also wraps and controls the release of pyraclostrobin. These results provided that microcapsulation could dramatically improve the photostability of pyraclostrobin, enhance the utilization efficiency and reduce the input of such agriculture chemicals.

### 2.5. Acute Toxicity against Zebra Fish 

During the experiment, no dead or abnormal behaviors were observed in the blank and solvent control groups. After poisoning by pyraclostrobin, a series of toxic symptoms of fish including acute irregular swimming, jumping out of the water, and flipping up and down were observed. When fish die, they finally turn over on their backs. The results of acute toxicity against zebra fish with different pyraclostrobin formulations are shown in Table 3. LC_50_ (24 h) values of 95% pyraclostrobin TC, 25% pyraclostrobin suspension concentrate (SC) and Pyr@CS-*g*-PDMAEMA were 0.0642, 0.0663, and 0.1020 mg/L, respectively. According to the pesticide toxicity classification standard suggested by the Chemical Pesticide Environmental Safety Evaluation Test Guidelines (GB/T 31270.12-2014), virulence of 95% pyraclostrobin TC, 25% pyraclostrobin SC and Pyr@CS-*g*-PDMAEMA on zebra fish were extreme toxicity, extreme toxicity, and high toxicity, respectively. Pyr@CS-*g*-PDMAEMA showed lower toxicity compared with that of control formulations on the first day. These results indicated that the microcapsulation of pyraclostrobin could reduce acute toxicity against zebra fish to some extent. However, the acute toxicity of Pyr@CS-*g*-PDMAEMA against zebra fish became comparative with that of control formulations over time. The LC_50_ (96 h) values of 95% pyraclostrobin TC, 25% pyraclostrobin SC and Pyr@CS-*g*-PDMAEMA were 0.0596, 0.0568, and 0.0674 mg/L, respectively. The acute toxicity test, on the other hand, verified the controlled release of pyraclostrobin from microcapsules. As time went on, most of the pyraclostrobin was released into the solution, resulting in similar toxicity to that of pyraclostrobin TC on the fourth day. The present study opens up the possibility that the acute toxicity of pesticides against fish can be reduced through elaborate process optimization of microcapsulation and precise regulation of pesticide release. 

## 3. Materials and Methods

### 3.1. Materials

Chitosan with the viscosity of 50–800 mPa·s and the average degree of deacetylation of 90% was purchased from Sinopharm Chemical Reagent Co., Ltd. (Shanghai, China). 2-(Dimethylamino) ethyl 2-methacrylate (DMAEMA) glutaraldehyde, and KPS were purchased from J&K Scientific Ltd. (Beijing, China). DMAEMA was purified by distillation under reduced pressure before use. Pyraclostrobin was obtained from Jiangsu Anpon Electrochemical Co., Ltd. (Huaian, China). Solvent oil S-200 was kindly provided by Jiangsu Hualun Chemical Industry Co., Ltd. (Yangzhou, China). Hydrochloric acid (HCl), acetone and other chemicals were all of analytical reagent and used as received without further purification. Ultrapure water used in all experiments was prepared using a MilliQ-50 SP reagent water system (Millipore Corporation, Bedford, MA, USA).

### 3.2. Synthesis of CS-g-PDMAEMA

The free radical polymerization technique was used to synthesise the CS grafted copolymer. Briefly, 3.0 g of CS was dissolved in 150 mL of water with 2% (*v*/*v*) acetic acid under constant stirring until it was completely dissolved. Then, the mixture was heated to 55 °C, and 12 mg of KPS was introduced into the flask while purged with nitrogen. The solution was agitated for 15 min, followed by the addition of 1.0 g of a DMAEMA monomer. The grafting reaction was carried out at 55 °C for 4 h under nitrogen atmosphere. Subsequently, the CS grafted copolymer was precipitated in an excess amount of acetone. DMAEMA homopolymers were removed by refluxing the raw products in acetone for 5 h, and then the purified copolymers were freeze-dried to a constant weight under vacuum to obtain the cotton-like final product, designated as CS-*g*-PDMAEMA.

### 3.3. Preparation of Pyraclostrobin Microcapsule

Microcapsules containing pyraclostrobin were prepared via an emulsion chemical cross-linking method. The above synthesized copolymer CS-*g*-PDMAEMA (0.2 g) was dispersed in 20 mL of 2% acetic acid solution containing 1% (*v*/*v*) Tween-80 to form a homogeneous solution at room temperature. To this mixture, a required amount of pyraclostrobin previously dissolved in solvent oil S-200 (50%, *w*/*w*) was added dropwise. The solution was emulsified to form oil-in-water (O/W) emulsion under constant stirring at 5000 rpm for 30 min using a high-speed stirrer (IKA Labortechnik, Staufen, Germany). Then, 1 mL of 0.1 M HCl and 1 mL of glutaraldehyde were added into the mixture. After continuing stirring for 10 min, the formed microcapsules were filtered and washed repeatedly with sufficient petroleum ether and water to remove the oil phase as well as an excess of surfactant and unreacted cross-linking agent. A pyraclostrobin encapsulated microcapsule was denoted as Pyr@CS-*g*-PDMAEMA, which was freeze-dried and stored in a desiccator for further characterization. The amounts of pesticide and glutaraldehyde were optimized to obtain satisfactory encapsulation efficiency (EE) of Pyr@CS-*g*-PDMAEMA. 

### 3.4. Loading Content and Encapsulation Efficiency 

For the determination of the pesticide loading content (LC), 20 mg of pesticide microcapsules was fully grinded and extracted with 100 mL of ethanol for 2 h by sonification at room temperature. Then, the microcapsules were centrifuged at 10,000 rpm for 10 min and fitted with a filter (0.45 μm pore size). The amount of pesticide released from the microcapsules in the test solution was analyzed by high performance liquid chromatography (HPLC, 1200 Series, Agilent, Santa Clara, CA, USA) equipped with a diode array detector. The LC (%) of pyraclostrobin and the encapsulation efficiency EE (%) of pyraclostrobin was calculated as follows: LC (%) = (weight of pesticide encapsulated in microcapsules/weight of microcapsules) × 100; EE (%) = (weight of pesticide encapsulated in microcapsules/initial weight of pesticide employed) × 100.

The operating parameters for HPLC determination were as follows: Venusil XBP-C_18_ reversed-phase column (5 μm × 4.6 mm × 250 mm, Bonna-Agela Technologies Inc., Tianjin, China); column temperature, 25 °C; mobile phase, (methanol: 0.2% formic acid aqueous solution (*v*/*v*) = 40:60), flow rate, 1.0 mL/min, injection volume, 10 μL; and diode array detector (DAD) signals, 295 nm. 

### 3.5. Characterization 

Fourier transform infrared (FT-IR) spectra of CS, pyraclostrobin, CS-*g*-PDMAEMA, and Pyr@CS-*g*-PDMAEMA were conducted on a spectrometer (NICOLET 6700, Thermo Scientific, Waltham, MA, USA) with a potassium bromide pellet. The spectra were collected in the range from 400 to 4000 cm^−1^ at a resolution of 4 cm^−1^. The morphological feature and particle size of pesticide microcapsules were characterized with scanning electron microscope (SEM, SU8000, Hitachi, Ltd., Tokyo, Japan) at an accelerating voltage of 10 kV. The ^1^H-NMR spectra were recorded on a Bruker Avance 300 spectrometer (Coventry, UK) operating at 300 MHz at room temperature. The samples (15 mg each) were dissolved in 0.7 mL of 1.0 M hydrochloric acid/deuterium oxide (D_2_O) mixed solvent (1:2, *v*/*v*) and placed in a 5 mm NMR tube. Chemical shifts were referenced to the solvent peak (4.7 ppm for D_2_O). 

In order to confirm the thermal stability of the grafted copolymers, thermogravimetric analysis (TGA) was performed with a Perkin Elmer Pyris Diamond (Woodland, CA, USA) from 20 to 550 °C at a heating rate of 15 °C/min under nitrogen atmosphere. TGA was also used to estimate the grafting percentage (G%) using the % residue obtained for chitosan and the grafted copolymers with the following equation: (G%) = (% residue for chitosan − % residue for copolymer/% residue for chitosan) × 100 [23].

### 3.6. In Vitro Release 

In addition, 20 mg of microcapsules were dispersed in 2.0 mL of release medium in dialysis bag (molecular weight cut off 8000–14,000), which was suspended in 200 mL of release medium in a dissolution tester (D-800LS, Tianjin University, Tianjin, China) at a rotation speed of 100 rpm at 30 ± 0.5 °C. The release medium was composed of phosphate buffer solution (PBS), ethanol and tween-80 emulsifier (140:59:1, *v*/*v*/*v*). The accumulative release profile of pyraclostrobin was calculated by determining the concentration of pyraclostrobin in the release medium at different times. One milliliter of the release medium was withdrawn at a given time intervals for HPLC assay followed by addition of 1 mL of fresh release medium to keep a constant volume. Different pH value (3.7, 5.7, and 7.3) and temperature (10, 25, and 35 °C) were set to investigate the stimuli-responsive release profiles. The accumulative release of pyraclostrobin was calculated according to the following equation [39]:

(1) $$E_{r}=\frac{V_{e}\sum_{i=0}^{n-1}C_{i}+V_{0}C_{n}}{m_{pesticide}}\times 100\%,$$

where *E_r_* was the accumulative release (%) of pyraclostrobin; *V_e_* was the volume of sample taken at a given time interval (*V_e_* = 1.0 mL); *V*_0_ was the volume of release solution (200 mL); *C_n_* (mg/mL) is the pyraclostrobin concentration in release medium at time *n*; *m_pesticide_* (mg) was the total amount of pesticide encapsulated in the microcapsules.

### 3.7. Photolysis Kinetics

Photostability of pesticides has profound impact on their effectiveness. The photostability experiment was conducted on a photochemical reactor (Beijing Precise Technology Co., Ltd., Beijing, China). Microcapsules containing 0.4 mg of pyraclostrobin were dispersed in 20 mL of 20% acetonitrile aqueous solution in a quartz tube, which was irradiated by a 500 W high-pressure mercury at room temperature under stirring rate of 100 rpm. 0.8 mL of the mixture was withdrawn at a certain time, and equal volume of the fresh media was added to conserve a constant volume. The sample was extracted by ultrasonic using 0.8 mL of acetonitrile at room temperature for 40 min and fitted with a filter (0.45 μm pore size) for HPLC analysis. For comparison, the photostability of pyraclostrobin technical concentrate was also studied, and the experimental procedure was the same as that for microcapsules. All the treatments were performed three times. The photolysis of pyraclostrobin was characterized by first-order kinetic equation. The calculation formula is as follows:

(2) $$\frac{dC}{dt}=kC,$$

(3) $$C_{t}=C_{0}e^{-kt},$$

where *k* was the photolysis rate constant, h^−1^; *C*_0_ was the initial concentration of pyraclostrobin in the solution; *C_t_* was the residual concentration at time *t*, mg/L. The half- life time (*t*_1/2_) of degradation was calculated when *C_t_* was *C*_0_/2.

### 3.8. Acute Toxicity of Pyraclostrobin against Zebra Fish 

In the present study, acute toxicity was conducted to evaluate the risk of pyraclostrobin against zebra fish by static tests according to the Organisation for Economic Co-operation and Development (OECD) guidelines [40] and the test guidelines on environmental safety assessment for chemical pesticides—Part 12: Fish acute toxicity test (GB/T 31270.12-2014) [41]. The experimental animal zebra fish was collected from the local fish market and stored under laboratory conditions. The experiment was conducted in the lab of Institute of Plant Protection, Chinese Academy of Agricultural Sciences, which was approved by the Ministry of Agriculture of the People’s Republic of China to provide technical service to conduct pesticide registration tests on environmental safety assessment against non-target organisms including earthworm, fish, bee, bird and so on. The number of qualification certificate is Environment-007 (24 January 2011). The experimental water was dechlorinated for 24 h with the pH value of 7.5~8.5. The dissolved oxygen content is maintained at above 5.8 mg/L, and experimental temperature was about (23 ± 2) °C. Zebra fish about 3–5 months old were used in this study. The feeding was stopped 24 h before the experiment and no feeding was given during the test. Ten tails of zebra fish were placed in a fish tank filled with 3 L aerated water containing different concentrations of pyraclostrobin microcapsule，pyraclostrobin SC and pyraclostrobin TC ranging from 0.05 to 0.12 mg/L. Control experiments with blank (aerated water) and solvent (acetone with dosage less than 0.05 mL/L) were performed under the same conditions. The experiment was repeated three times. The death rate and poisoning symptoms of zebra fish were observed and recorded at 24, 48, 72, and 96 h, respectively. The LC_50_ values (mg/L) were calculated by software SPSS13.0 (SPSS Inc., Chicago, IL, USA). 

## 4. Conclusions

In conclusion, CS-*g*-PDMAEMA microcapsules loaded with pyraclostrobin were fabricated via an emulsion chemical cross-linking method with glutaraldehyde as the cross-linking agent. The pyraclostrobin-loaded microcapsules with drug loading of 18.79% showed pH- and thermo responsive release. The cumulative release of pyraclostrobin was higher in acidic medium in comparison with that in basic and neutral solutions. An increase of pesticide release with increasing temperature was obviously observed. Compared to free pyraclostrobin, microcapsulation could dramatically improve its photostability under ultraviolet light irradiation, which provides an alternative strategy for such photo unstable pesticides. Consequently, the utilization efficiency of pyraclostrobin can be enhanced and the input of such agriculture chemical can be decreased. Moreover, microcapsulation could reduce the acute toxicity of pyraclostrobin against zebra fish to some extent on the first day, and reached a similar toxicity to that of pyraclostrobin TC over time. This stimuli-responsive pesticide delivery system may find potential application in sustainable plant protection. 

## Figures and Tables

**Table 1:** The loading content (LC) and encapsulation efficiency (EE) of pyraclostrobin into CS-*g*-DMAEMA microcapsules.

Entry	CS-*g*-DMAEMA (g)	Pesticide (g)	Glutaraldehyde (mL)	LC (%) *^a^*	EE (%) *^a^*
1	0.2	0.2	0.6	17.09 ± 1.18	41.01 ± 2.83
2	0.2	0.2	0.8	19.22 ± 0.30	47.57 ± 0.78
3	0.2	0.2	1.0	18.79 ± 0.41	64.51 ± 1.38
4	0.2	0.3	1.0	20.52 ± 0.49	54.72 ± 1.30
5	0.2	0.1	1.0	9.20 ± 1.15	48.40 ± 6.04

*^a^* Mean value ± SD (*n* = 3).

**Table 2:** Photolyses of pyraclostrobin TC and microcapsules under high-pressure mercury lamp.

Analytes *^a^*	First-Order Kinetic Equation			
	*C_t_* = *C*_0_*e^−kt^*	*k* (h^−1^)	*R*²	*t*_1/2_ (h)
95% Pyr TC	*y* = 21.13*e*^−0.22*x*^	0.22	0.915	3.2
Pyr@CS-*g*-PDMAEMA	*y* = 15.84*e*^−0.03*x*^	0.03	0.918	23.1

*^a^* Pyr: pyraclostrobin, TC: technical concentrate.

**Table 3:** Acute toxicity of pyraclostrobin against zebra fish.

Analytes	Exposure Time (h)	LC_50_ (mg/L)	95% Confidence Interval	*R*²	Equation
95% Pyr TC	24	0.0642	0.0587–0.0775	0.9024	*y* = 15.4958 + 8.8019*x*
	48	0.0618	0.0566–0.0694	0.8828	*y* = 16.8795 + 9.8240*x*
	72	0.0597	0.0558–0.0635	0.9931	*y* = 22.8589 + 14.5894*x*
	96	0.0596	0.0560–0.0651	0.9899	*y* = 23.3577 + 14.9872*x*
25% Pyr SC	24	0.0663	0.0594–0.0770	0.9782	*y* = 14.0210 + 7.6539*x*
	48	0.0612	0.0540–0.0688	0.9648	*y* = 14.3705 + 7.7241*x*
	72	0.0586	0.0519–0.0643	0.9717	*y* = 16.5114 + 9.3429*x*
	96	0.0568	0.0494–0.0624	0.9678	*y* = 16.3531 + 9.1147*x*
Pyr@CS-*g*-PDMAEMA	24	0.1020	0.0865–0.1747	0.7266	*y* = 9.7209 + 4.7611*x*
	48	0.0895	0.0670–0.0962	0.9716	*y* = 8.1220 + 2.9785*x*
	72	0.0674	0.0321–0.0786	0.9278	*y* = 11.5447 + 5.5876*x*
	96	0.0674	0.0321–0.0786	0.9278	*y* = 11.5447 + 5.5876*x*
